# Commentary: More is better: Hybrid and parallel extracorporeal membrane oxygenation circuits

**DOI:** 10.1016/j.xjtc.2021.03.002

**Published:** 2021-03-04

**Authors:** Francis D. Pagani

**Affiliations:** Department of Cardiac Surgery, University of Michigan, Ann Arbor, Mich

**Figure:**
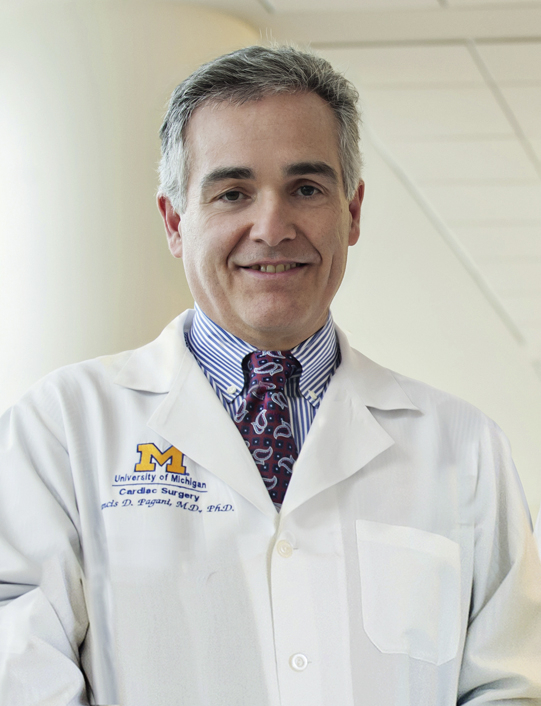
Francis D. Pagani, MD, PhD

**Central Message**

Hybrid and parallel extracorporeal membrane oxygenation circuits offer important options in clinical scenarios of inadequate perfusion or oxygenation.

See Article page 77.

The application of extracorporeal membrane oxygenation (ECMO) for treatment of respiratory and/or cardiac failure has increased over the past decade.^1^ Expansion of indications and inclusion of higher-risk patients has increased the number of challenging clinical scenarios in which limitations in oxygen delivery with conventional ECMO circuits are encountered during support. In this issue of the *JTCVS Techniques*, Shah and colleagues^2^ from the University of Maryland review the use of hybrid and parallel ECMO circuits in clinical scenarios in which standard venoarterial (VA) or venovenous (VV) ECMO circuits fail to provide adequate oxygen delivery or perfusion and provide a number of important management and technical observations and recommendations.

Importantly, the authors emphasize 2 principals: that clinical situations necessitating hybrid or parallel circuits are infrequent and that optimal patient management and technical application of conventional ECMO circuits is paramount before considering hybrid or parallel ECMO circuits. Appropriate cannula positioning, optimal hemodynamic and respiratory management, appropriate left ventricular venting, and potential conversion to central cannulation are important key tenets determining successful support with conventional ECMO circuits.

Shah and colleagues describe several specific hybrid circuits and the clinical scenarios in which hybrid circuits are useful. With respect to patients supported with VA ECMO who experience upper body hypoxia due to concomitant respiratory failure and competition of ECMO flow with native cardiac output (ie, north-south or Harlequin syndrome), the authors describe the value of the venoarteriovenous ECMO circuit. In this scenario, an additional venous cannula positioned in the right side of the circulatory system returns oxygenated blood from the ECMO circuit to the heart to reduce the proportion of deoxygenated blood being ejected by the native heart to resolve the upper body hypoxia. Importantly, the authors review strategies to assess and manage arterial flow diversion in this hybrid circuit, specifically applying resistance to the arterial outflow to the venous system to prevent overflow. For patients initially supported for respiratory failure with VV ECMO, progressive hypoxia or cardiac failure may lead to the addition of an arterial cannula to establish a VVA ECMO circuit, where both cannulas now in the right side function as venous return to the ECMO circuit.

In addition to the hybrid circuits, the authors also discuss the use of parallel circuits. For patients with high cardiac output and ongoing hypoxia or with evidence of inadequate perfusion a second or parallel ECMO circuit could provide additional capacity for oxygenation and flow. The use of parallel circuits can be applied in the scenarios of both VV ECMO and VA ECMO support. The authors raise appropriate concerns for recirculation in the setting of parallel VV ECMO circuits. In the setting of parallel VA ECMO circuits, capture of too much of the native flow through the heart and lungs could increase the risk of thrombosis.

Shah and colleagues have provided readers with a valuable review of hybrid and parallel ECMO circuits, including an excellent diagrammatic presentation and summary of clinical outcomes. Application of these techniques, although infrequently needed, is an essential and required skill set for centers managing patients supported with ECMO.
